# A New Technique of Oophoropexy: Folding and Fixating of Utero-Ovarian Ligament to Round Ligament in a Patient with Reccurrent Ovarian Torsion

**DOI:** 10.1155/2019/7647091

**Published:** 2019-11-21

**Authors:** Mehmet Obut, Uğur Değer

**Affiliations:** ^1^Department of Obstetrics and Gynecology, Health Sciences University, Diyarbakır Gazi Yaşargil Training and Research Hospital, Diyarbakır, Turkey; ^2^Department of Obstetrics and Gynecology, Dicle Memorial Hospital, Diyarbakır, Turkey

## Abstract

**Introduction:**

Most cases of ovarian torsion occur in the reproductive age and many are related to ovarian and paraovarian masses. If it occurs without any subtle anatomic etiology or ovarian and paraovarian masses, recurrence of ovarian torsion is more likely. Recurrent left ovarian torsion is much less common than recurrent right ovarian torsion. The authors describe a rare case with their new ovarian fixation technique.

**Case and Method:**

A 21-year-old female patient with polycystic ovarian syndrome had a 7th recurrence of left ovarian torsion. Although ovarian fixation was performed in addition to detorsion at the 5th and the 6th laparoscopic surgeries, it had failed. Due to recurrence ovarian torsion after ovarian fixation, the authors performed a different technique:folding the utero-ovarian ligament which folded on itself. The distal part of the utero-ovarian ligament with the ovary was both fixed to the proximal part of the round ligament which was adjacent to the uterus.

**Conclusion:**

Although a wide range of oophoropexy methods have been described in the literature, the best method remains a matter for debate. This technique can be performed even when the ovarian tissue is necrotic. Because of this, we think that this technique is useful and effective technique.

## 1. Introduction

Ovarian torsion is a gynecological emergency since the blood circulation to the ovary is disrupted due to the rotation of the ovary alone or along with the uterine tubes around its ligaments and may progress to ovarian necrosis if not corrected promptly.

Ovarian torsion represents about 2.5–5% of all gynecological emergencies in women of reproductive age [1, 2]. The risk of ovarian torsion rises if the ovarian mass increases for any pathologic reason (e.g., polycystic ovarian syndrome and ovarian teratoma) or if the ligaments are longer than normal [3, 4]. More than 80% of patients with ovarian torsion had ovarian masses of 5 cm or larger, indicating that the primary risk in ovarian torsion is an ovarian mass, contrarily premenarchal girl with ovarian torsion are more commonly found with a normal ovary [3–5].

Both the diagnosis and treatment of ovarian torsion is carried out via laparoscopy in most cases because it is a minimally invasive technique [6]. In the past, ovarian torsion correction without salpingo-oophorectomy was considered a risk factor for thromboembolic events, but it is now known that the risk of thromboembolic events is low and that ovarian tissue typically later regains normal follicular activity [7]. The rate of recurrent ovarian torsion is low, and the frequency of the left ovarian torsion is two times less than that of right ovarian torsion [8]. A recurrence of ovarian torsion is more likely if the previous torsion occurred in the absence of any adnexal mass [7]. Patients who have experienced a previous episode of ovarian torsion typically more easily todiagnose their recurrent ovarian torsion compared to their first episode [9]. The best method of patient selection for the oophoropexy (ovarian fixation) procedure is still being debated [8]. Oophoropexy is a surgical procedure carried out to protect the fertility of the patient and to reduce the risk of future ovarian torsion, but the debate is still on-going regarding the best correction method [6, 10]. In this study, we report an extraordinary case who suffered from seven repeated left ovarian torsions and had undergone four laparoscopic detorsions and three laparoscopic detorsions with oophoropexy procedures in the last year. To our knowledge and based on literature review, this is the first case with these many recurrences of left ovarian torsion.

## 2. Case and Method

A 21-year-old female patient with the polycystic ovarian syndrome (PCOS) who denied any past sexual activity had undergone laparoscopic ovarian left detorsion six times before; oophoropexy was performed during the last two surgeries. The patient presented to the emergency department with pain in the lower left quadrant of the abdomen approximately 20 days after her last operation. The patient reported that her pain was the same as she had experienced during her previous left ovarian torsion. The patient also had menstrual irregularty and hirsutism (Ferriman–Galloway score was 10). She had no vomiting, nausea, fever, diarrhea, or dysuria. Left lower quadrant tenderness and rebound tenderness were present during the patient's physical examination. There were no abnormal laboratory results. The ultrasonographic examination revealed that the right ovary's appearance was consistent with PCOS. The left ovary was bigger than normal, and blood flow to the left ovary was not visualised.

### 2.1. Patient's History

12 January 2018 first laparoscopic left ovarian detorsion was performed, and the left ovary was torsed 3x counterclockwise around IPL.

2 February 2108 second laparoscopic detorsion was performed due to 2 counterclockwise torsioned left ovary and laparoscopic detorsion was performed.

25 March 2018 third laparoscopic detorsion was performed given that the left ovary torsed at 4x counterclockwise.

12 June 2018 fourth detorsion was performed for 4x counterclockwise torsion.

19 September 2018 fifth laparoscopic left ovarian detorsion and utero-ovarian ligament plication was performed, given that the left ovary was torsed 3x counterclockwise.

22 December 2018 sixth laparoscopic of the left ovarian detorsion with utero-ovarian ligament plication and ovarian fixation to the round ligament was performed.

**Figure 1:**
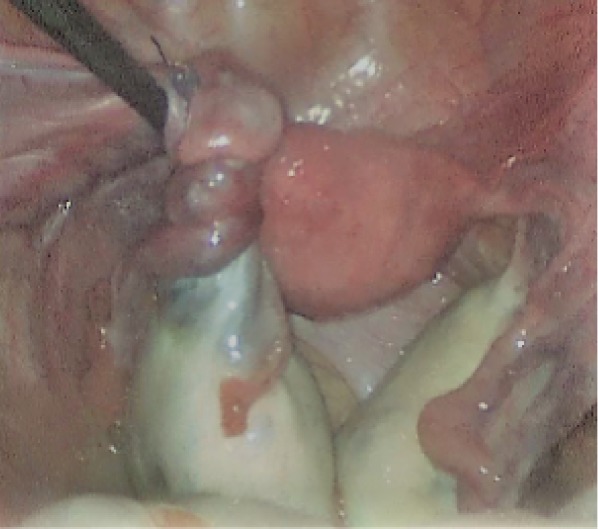
Left ovary being untwisted with a blunt instrument.

**Figure 2:**
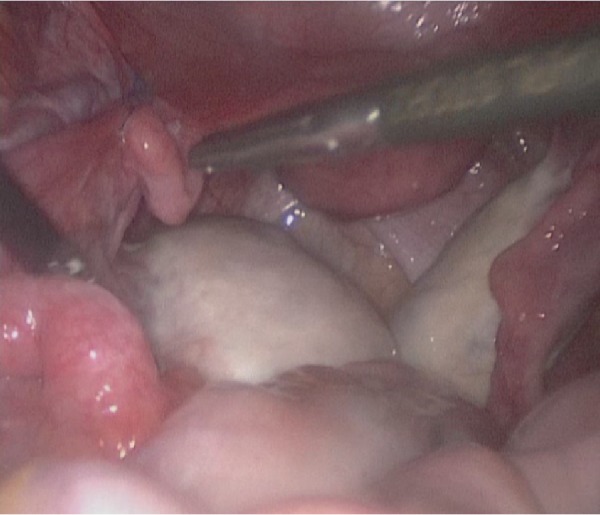
Markedly elongated left utero-ovarian ligament compare to right side.

**Figure 3:**
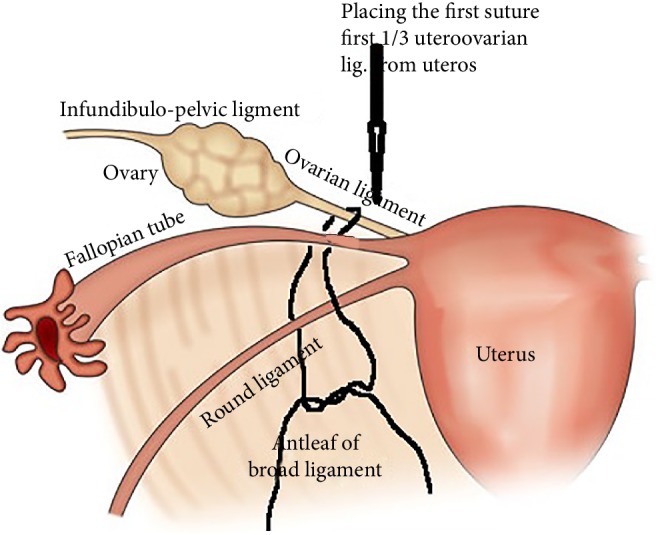
Placing of first suture.

**Figure 4:**
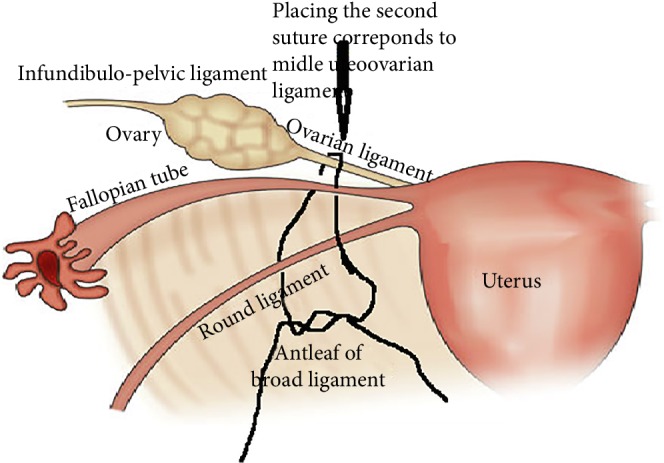
Placing of second suture.

**Figure 5:**
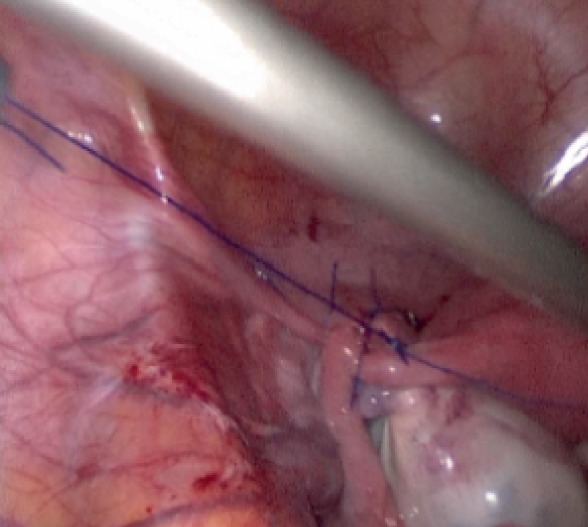
Implementation of last suture.

**Figure 6:**
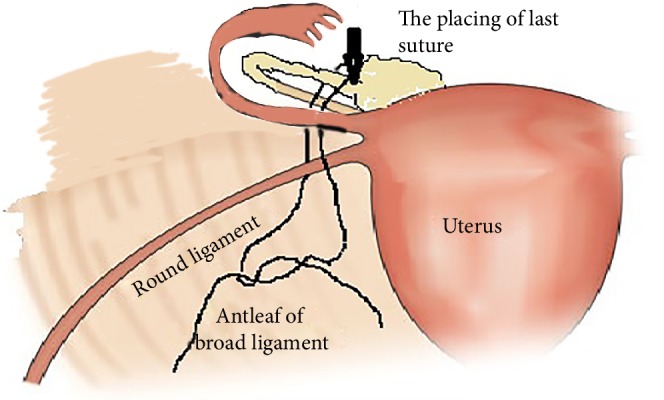
Placing of last suture.

**Figure 7:**
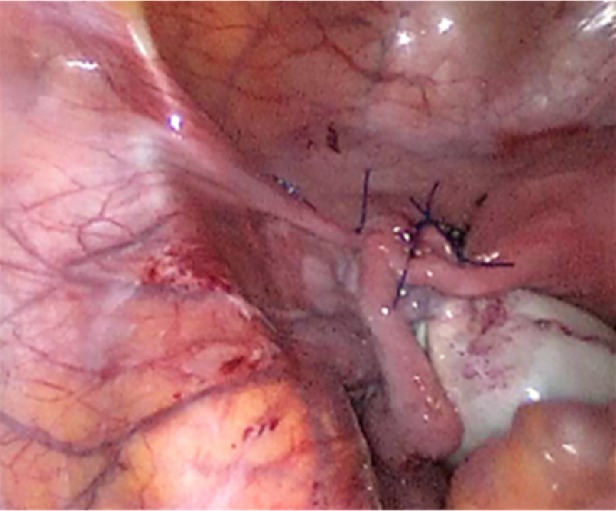
Position of uterus tubes and ovaries after fixation procedure completed.

18 January 2019 the patient's last laparoscopic left ovarian detorsion was performed. In surgery left ovarian torsion occurred 5 times counterclockwise around the IPL. There was no significant color change (Figure 1). The left ovary was untwisted with blunt instruments. There were nonabsorbable sutures used from the previous surgeries for ovarian fixation (Figure 2). Ovarian fixation to the sacrouterine ligament and the UOL plication had been performed in the last two surgeries when ovarian torsion occurred. For this reason we decided to implement a different technique that would be better for this patient. In this technique, a 1/0 prolene suture was used.****The first suture application is that the needle was passed through the round ligament, which corresponds to the first 1/3 of the utero-ovarian ligament from the uterus. Then it was passed through the avascular space under the tube and the utero-ovarian ligament. After that, the suture was redirected through avascular space under the tube and the inferior part of the round ligament and the suture was knotted (Figure 3). The second suture repeats as in the first suture, which corresponds to the middle of the utero-ovarian ligament (Figure 4). The third suture application the needle was passed through the round ligament adjacent to the cornual part of the uterus, then exited from the avascular space under the tuba, such that the ovary and utero-ovarian ligament were brought closer to the cornual region of the uterus. The needle was passed through the distal part of the utero-ovarian ligament adjacent to the ovary after the suture was redirected through the avascular space under the tuba uterina and the round ligament, and then the suture was knotted (Figures 5, 6, 7). The postoperative period was uneventful. In the ultrasound examination of the patient at 2 and 4 months postoperatively, both the ovaries were polycystic, the left and the right ovary was 5 × 4 × 4 and 5 × 4 × 4 cm in size and the blood flow to both ovaries were normal.

## 3. Discussion

To our knowledge, this is the first case in the English literature with seven different reoccurrences of the left ovarian torsion following laparoscopic detorsion in the past year, with the last three procedures, including oophoropeies. During the seventh episode, both ovaries were polycystic; the left ovary was the 7x6x6 cm in size, while the right ovary was 5x4x4 cm. There was a slight change in the color of the left ovary, but no color change was seen on the right ovary. The left utero-ovarian and the infundibulopelvic ligaments were longer than those on the right. Typically, the infundibulopelvic ligament of the right ovary is longer in most patients, and the sigmoid colon decreases the mobility of the left ovary. Therefore, the possibility of torsion of the right ovary is more likely than torsion of the left ovary [11]. In our case, however, all torsion episodes occurred in the left ovary. The reason for the left ovary torsion in our patient may have been that the left ligaments were much longer than the right ligaments in her case. Patients who have benign adnexal masses, especially cystic teratomas, are more likely to experience torsion than patients with endometriomas or malignant adnexal masses. Because adhesions and invasions are generally associated with endometriomas and malignant adnexal masses, and they decrease the possibility of torsion. The frequency of adnexal torsion increases during pregnancy, which is attributed to the corpus luteum [12, 13]. When the diagnosis of ovarian torsion is made, surgery is the preferred treatment and should not be delayed if the patient desires to preserve ovarian function. Today, laparoscopy is the preferred treatment method because it is minimally invasive [14, 15]. Oophoropexy is primarily reserved for the prevention of ovarian torsion recurrence in patients in whom the possibility of a recurrence is high.

According to the literature, the risk of recurrence of ovarian torsion with a reversible etiology (e.g., ovarian cyst) and/or torsion that occurs for the first time is low and should be treated with simply untwisting the ovary because of the theoretical risk that an oophoropexy or any other prophylactic treatment can prevent a recurrence [7, 16, 17]. However, the literature also revealed that recurrent and asynchronous bilateral ovarian torsion episodes are also more likely when ovarian torsion occurs in a normal or polycystic ovary and in such cases there is a tendency to perform oophoropexy in the literature [7, 16, 18].

Because of the theoretical risk that oophoropexy may disrupt blood circulation and damage the tubo-ovarian connection or tubal function, an analysis of the risks and benefits should be carried out in each patient before surgery. Çelik et al. reported that one in nine of their cases who underwent bilateral oophoropexy with nonabsorbable sutures experienced ovarian atrophy [17].

Several oophoropexy techniques have been reported, such as fixation of the ovary to the pelvic side wall, the posterior uterine wall, the sacro-uterine ligament, the posterior abdominal wall, and the ligamentum teres uteri both with and without plication of the utero-ovarian ligament [6, 10, 12, 19, 20]. The best management strategy of recurrent ovarian torsion is still under debate, and no clear answer exists regarding patient selection, timing, the preferred surgical method, and whether the procedure should be performed bilaterally or unilaterally [7, 17]. During our patient's seventh torsion episode, the left ovarian appearance was slightly changed, and there were not any ischemic-hemorrhagic signs, so we performed the oophoropexy after the ovarian detorsion in the same surgery. Generally, ischemic, and oedematous ovaries are common with ovarian torsion; therefore, the ovarian tissue can be fragile. In these cases, it is recommended that the oophoropexy be postponed because of the fragile tissue that might cause suture instability and failure [6, 10]. Bilateral oophoropexy should be considered in case of asynchronous ovarian torsion, particularly with persistent etiology [9]. Oophoropexy or ovarian fixation does not perculade ovarian retorsion. 9.5% retorsion after oophoropexy is reported by Tsafrir et al. [22].To our knowledge, for long time various oophoropexy methods' results have not been published. Selected methods of oophoropexy should be individualized. Elongated utero-ovarian ligament, age, history of previous ovarian torsion, application of oophoropexy method, with or without adnexal mass, the status of ovarian tissue, and pregnancy status should be considered [16, 14, 23]. Most surgeons, especially if the uteroovarian ligament is too long, prefer an utero-ovarian plication as the first step. The fibrous ligaments are less susceptible to the ischemic event [21, 22]. It is important that, regardless of the method chosen, oophoropexy nonabsorbable sutures must be used [7].

In conclusion with this technique oophoropexy is generally possible even with an ischemic and hemorrhagic ovary because of using the utero-ovarian and round ligaments. As described above with this technique by folding the ligament, finally, the length of the utero-ovarian ligament is shortened, a wide ovarian pedicle is obtained, fixed and stabilized to the round ligament. Unlike ovarian fixation to a pelvic sidewall or posterior abdominal wall, there is no risk of vascular and ureter injury.
